# Non-syntenic genes drive RTCS-dependent regulation of the embryo transcriptome during formation of seminal root primordia in maize (*Zea mays* L.)

**DOI:** 10.1093/jxb/erw422

**Published:** 2016-11-10

**Authors:** Huanhuan Tai, Nina Opitz, Andrew Lithio, Xin Lu, Dan Nettleton, Frank Hochholdinger

**Affiliations:** 1 Institute of Crop Science and Resource Conservation, Crop Functional Genomics, University of Bonn, 53113 Bonn, Germany; 2 Department of Statistics, Iowa State University, Ames, IA 50011–1210, USA; 3 Experimental Medicine and Therapy Research, University of Regensburg, 93053 Regensburg, Germany

**Keywords:** Embryo, primordia, RNA-Seq, *rtcs*, seminal root, transcriptome, *Zea mays*.

## Abstract

Seminal roots of maize are pivotal for early seedling establishment. The maize mutant *rootless concerning crown and seminal roots* (*rtcs*) is defective in seminal root initiation during embryogenesis. In this study, the transcriptomes of wild-type and *rtcs* embryos were analyzed by RNA-Seq based on histological results at three stages of seminal root primordia formation. Hierarchical clustering highlighted that samples of each genotype grouped together along development. Determination of their gene activity status revealed hundreds of genes specifically transcribed in wild-type or *rtcs* embryos, while K-mean clustering revealed changes in gene expression dynamics between wild-type and *rtcs* during embryo development. Pairwise comparisons of *rtcs* and wild-type embryo transcriptomes identified 131 transcription factors among 3526 differentially expressed genes [false discovery rate (FDR) <5% and |log_2_Fc|≥1]. Among those, functional annotation highlighted genes involved in cell cycle control and phytohormone action, particularly auxin signaling. Moreover, *in silico* promoter analyses identified putative RTCS target genes associated with transcription factor action and hormone metabolism and signaling. Significantly, non-syntenic genes that emerged after the separation of maize and sorghum were over-represented among genes displaying RTCS-dependent expression during seminal root primordia formation. This might suggest that these non-syntenic genes came under the transcriptional control of the syntenic gene *rtcs* during seminal root evolution. Taken together, this study provides first insights into the molecular framework underlying seminal root initiation in maize and provides a starting point for further investigations of the molecular networks underlying RTCS-dependent seminal root initiation.

## Highlight

RTCS regulates the expression of non-syntenic genes and genes associated with phytohormone action, transcription factors, and cell cycle regulators to control seminal root initiation during embryogenesis in maize.

## Introduction

Specialized root systems in plants are vital for growth and adaptation because they provide mechanical support, mediate water and nutrient uptake, and interact with the rhizosphere (reviewed in Hawkes *et al.*, 2007; Lynch, 2013; Villordon *et al.*, 2014). The complex maize root system comprises roots that are laid down during embryogenesis and post-embryonic roots that are formed after germination. The embryonic root system includes a primary root and a variable number of seminal roots, while shoot-borne and lateral roots are formed post-embryonically (reviewed in Hochholdinger *et al.*, 2004; Yu *et al.*, 2016). Primary roots are initiated at the basal pole of the embryo and emerge 2–3 days after germination. Seminal roots are initiated at the scutellar node between 22 and 40 days after pollination and emerge in germinating seeds soon after the primary root (Sass, 1977; Erdelska and Vidovencova, 1993). Seminal roots are formed primarily by dorsal primordia (Salvi *et al.*, 2016). The number of seminal roots in maize varies within a genotype and between different genotypes, and ranges from 0 to 13 (Erdeska and Vidovencova, 1993; Burton *et al.*, 2013; Tai *et al.*, 2016).

While maize does form seminal roots, its close relative sorghum (*Sorghum bicolor*) does not (Singh *et al.*, 2010). The number of seminal roots has increased during maize domestication in landraces and modern varieties compared with their ancestor teosinte, suggesting that seminal root number has probably been selected inadvertently as an adaptive trait during maize domestication (Burton *et al.*, 2013, Tai *et al.*, 2016). Seminal roots are essential in the establishment and early development of maize seedlings (Sanguineti *et al.*, 1998; Lynch, 2011). Moreover, at later developmental stages, seminal root length and number are positively correlated with shoot biomass at low phosphorus levels (Zhu *et al.*, 2006). Furthermore, seminal root length correlates with grain yield under both low and high nitrogen levels (Abdel-Ghani *et al.*, 2015). Other seminal root traits such as number, angle, and diameter have been considered in designing hypothetical root ideotypes optimized for maize adaptation to a range of environmental stresses and increased sustainability of water and nutrient acquisition (Lynch, 2013). QTL mapping studies have suggested that a small number of major loci controls seminal root formation (Tuberosa *et al.*, 2002; Hund *et al.*, 2004; Zhu *et al.*, 2006; Burton *et al.*, 2013). This would facilitate a precise manipulation of root ideotypes to obtain optimized seminal root traits (Lynch, 2013; Salvi *et al.*, 2016).

In maize, two monogenic mutants defective in seminal root initiation have been described: *rootless concerning crown and seminal roots* (*rtcs*) and *rootless with undetectable meristems 1* (*rum1*). These two genes have been co-mapped with the two major QTLs controlling seminal root number (Salvi *et al.*, 2016) and are key components of auxin signaling (Taramino *et al.*, 2007; von Behrens *et al.*, 2011). The *rum1* gene encodes the monocot-specific Aux/IAA10 protein (von Behrens *et al*., 2011), while *rtcs* encodes a member of the plant-specific LATERAL ORGAN BOUNDARIES DOMAIN (LBD) transcription factor family (Taramino *et al.*, 2007; Majer *et al.*, 2012). In maize, the LBD family comprises 43 members (Majer and Hochholdinger, 2011). LBD proteins have crucial functions in defining lateral organ boundaries and are involved in various root-related developmental processes, including lateral root formation in Arabidopsis (Péret *et al.*, 2009), lateral and shoot-borne root formation in rice (Inukai *et al.*, 2005), and shoot-borne and seminal root formation in maize (Taramino *et al.*, 2007). LBD proteins contain a characteristic N-terminal LOB domain (Shuai *et al.*, 2002) that can bind to the LBD motif (5′-GCGGCG-3′) in promoters of target genes (Husbands *et al.*, 2007). RTCS can bind to the LBD motifs of the auxin response factor *arf34*, and the ARF34 protein can, vice versa, bind to *rtcs* (Majer *et al.*, 2012).

In the present study, RTCS-mediated transcriptional regulation of maize seminal root initiation during embryogenesis was studied by RNA-Seq. The objective of this study was to identify genes regulated by RTCS during maize embryogenesis and to gain novel insights into the molecular framework underlying seminal root primordia formation.

## Materials and methods

### Plant material and growth conditions

Surface-sterilized seeds of maize families segregating 3:1 for wild-type versus mutant *rtcs* seedlings were germinated in distilled water in paper rolls (Hetz *et al.*, 1996) and grown for 16 h in light at 28 °C and 8 h in dark at 22 °C at a constant humidity of 60% in a growth chamber. Seedlings at 10 d old were phenotyped and genotyped according to Xu *et al.* (2015) to select homozygous *rtcs* and homozygous wild-type siblings. Subsequently, homozygous wild-type and mutant seedlings were transferred to soil pots in a growth chamber and grown under the same conditions as the paper rolls. After selfing, embryos were harvested from homozygous wild-type and mutant *rtcs* plants at 25, 30, and 35 d after pollination. Identification of homozygous wild-type and mutant plants from segregating families ensured a very close genetic relationship of these genotypes. The segregating families from which these plants were selected had been previously selfed for more than seven generations and were thus highly isogenic.

### Histology of maize embryos

Embryos were fixed in 4% paraformaldehyde for 12 h at 4 °C and subsequently embedded in paraffin as described by Lim *et al.* (2000). Cross-sections of 14 µm were prepared with a Leica 2035 biocut-microtome (Leica, Nussloch, Germany). Sections were then deparaffinized and stained with Safranin O (AppliChen, Darmstadt, Germany) and Fast Green (Sigma-Aldrich, Taufkirchen, Germany) as previously described (Hetz *et al.*, 1996). Stained embryo sections were examined under a Zeiss-Axioskop HBO 100W/2 microscope (Zeiss, Jena, Germany) and photographed using a bright field camera (PixCell IIe System, Krefeld, Germany).

### Embryo RNA isolation and RNA-Seq

At 25, 30, and 35 d after pollination, embryos of similar size of the two genotypes were subjected to RNA-Seq analyses. Pools of 10 embryos were sampled per biological replicate. Four independent biological replicates were analyzed for each genotype and developmental stage. After harvest, embryos were immediately frozen in liquid nitrogen and stored at −80 °C until RNA isolation. Total RNA was extracted with the RNeasy Plant Mini Kit (Qiagen, Venlo, Netherlands). RNA integrity and quality were assessed by agarose gel electrophoresis and on an Agilent RNA 6000 Nano Chip in an Agilent 2100 Bioanalyzer (Agilent Technologies, Santa Clara, CA, USA). High-quality samples with RIN (RNA integrity number) values >8 were subjected to RNA-Seq (Illumina, San Diego, CA, USA). cDNA libraries were prepared as described by the manufacturer. Finally, the Illumina HiSeq 4000 platform was used to generate 100-bp paired-end reads.

### Processing and mapping of reads

Raw sequencing reads were trimmed by adapter removal. Low-quality reads containing ˃50% low-quality bases were excluded. Read mapping to the maize reference genome sequence was performed with CLC Genomics Workbench software (Version 8.0.1; https://www.qiagenbioinformatics.com/products/clc-genomicsworkbench/). Reads were first mapped to the maize B73 reference genome sequence (RefGen_v2; ftp://ftp.gramene.org/pub/gramene/maizesequence.org/release-5b/assembly/). If at least 90% of a read matched with 90% similarity to the reference, it was considered as mapped. Stacked reads that share the same start and end coordinate were considered as one read. Gaps up to 50 kb were allowed within reads to span introns. Mapped reads were further projected to the filtered gene set (FGS v2; Release 5b; ftp://ftp.gramene.org/pub/gramene/maizesequence.org/release-5b/filtered-set/). Only reads that mapped to unique positions in the filtered gene set with 80% of their bases displaying 90% similarity to the reference were selected for subsequent expression analyses. RNA-Seq data have been deposited in the NCBI sequencing read archive (SRA; http://www.ncbi.nlm.nih.gov/sra) at AC: SRP079373.

Sample relationships were analyzed by a Principal component analysis (PCA) and hierarchical clustering. PCA was conducted by using the prcomp function in R with default settings. Hierarchical clustering of all samples was generated based on Pearson correlations in the CLC Genomics Workbench package.

### Statistical procedures to determine gene activity

The transcriptional activity status of all genes (active/inactive) in wild-type and *rtcs* embryos at each developmental stage was determined using a generalized linear mixed model with a negative binomial response. The log of the mean was assumed to be a linear combination of fixed and random effects, plus sample and gene-specific normalization factors (described below). Each combination of genotype and stage was represented by a fixed effect, and random effects accounting for additional variation from sequencing lanes were also included. The log of the TMM normalization factor (Robinson and Oshlack, 2010) was added to normalize across samples, and a smooth function of gene length and GC content was used to normalize across genes.

The vector of fixed effects for each gene was assumed to be a draw from a multivariate normal distribution with an unknown and unrestricted mean and an unknown diagonal variance–covariance matrix. Within a gene, the log of the negative binomial dispersion parameter was assumed to be constant and a draw from a normal distribution with unknown mean and variance. The random effects were assumed to follow gamma distributions, where the parameters for the lane effects were specified to create a vague distribution. An empirical Bayes procedure via the R package ‘ShrinkBayes’ (Van De Wiel *et al.*, 2012) was used to estimate the unknown parameters, and to approximate the posterior distribution for the fixed effect associated with the gene (g), genotype (t), and stage (s) using the integrated nested Laplace approximation (Rue *et al.*, 2009).

Activity of a gene was determined by computing *P*_gts_(*T*), the posterior probability that the fixed effect for gene (g), genotype (t), and stage (s) was larger than a given threshold *T*. A gene (g) was called active for genotype (t) and stage (s) if *P*_gts_(*T*)>0.5 and otherwise inactive. This method classifies genes as active or inactive based on the posterior distribution of fixed effects considering raw read count, sequencing differences from sample to sample, gene length, and GC content differences, and therefore is more accurate than classifications based only on a single raw read count threshold applied to all genes.

### Statistical analysis of differential gene expression

To determine differential expression, only genes with a minimum of five mapped reads in all four biological replicates of at least one genotype were taken into account. Gene expression was normalized as FPKM (fragments per kilobase of exon model per million mapped reads) values. The R package limma based on linear models (Ritchie *et al.*, 2015) was used to identify differentially expressed genes in pairwise contrasts. The model takes the lane effect into account, considering it as a random effect. The number of differentially expressed genes was controlled by FDR<5% and |log_2_Fc|≥1. Euclidean algorithm-based K-means clustering was performed to generate the expression clusters of gene expression dynamics along three stages using the OmicShare tools (www.omicshare.com/tools). The eight clusters of the identified gene dynamic expression were based on the formula 3^(*t*−1)^–1 for theoretical possible dynamic expression patterns. ‘*t*–1’ indicates two transitions across three time points in this study. The number ‘3’ indicates the three expression patterns of a gene: increase, decrease, or stay the same between consecutive time points. The number ‘1’ indicates the cluster of which the expression of genes has no change through time points. Genes were assigned to the different clusters with FDR<5% and |log2Fc|≥1 for each of the pairwise comparisons to identify robust expression patterns.

### Functional annotation of genes and metabolic pathway analyses

The function of genes was annotated using MapMan (http://mapman.gabipd.org/web/guest/mapman) based on the functional annotation file ZmB73_5b_FGS_2011 and their homologs in Arabidopsis and rice, and a list of maize curated genes (Thimm *et al.*, 2004). Identification of significantly over- or under-represented categories was determined by a *χ* ^2^ test (*P*<0.01) with Yate’s continuity correction. A category was declared over-represented if significantly more genes than expected were assigned to it. The expected gene number in each of the 32 major annotated categories was calculated based on the distribution of all expressed genes to the categories. Annotation of transcription factors (TFs) and assignment into TF families was based on the Plant Transcription Factor Database (v3.0; http://planttfdb.cbi.pku.edu.cn/; Jin *et al.*, 2013).

## Results

### Histological analyses of seminal root primordia formation in wild-type and *rtcs* embryos

**Fig. 1.**
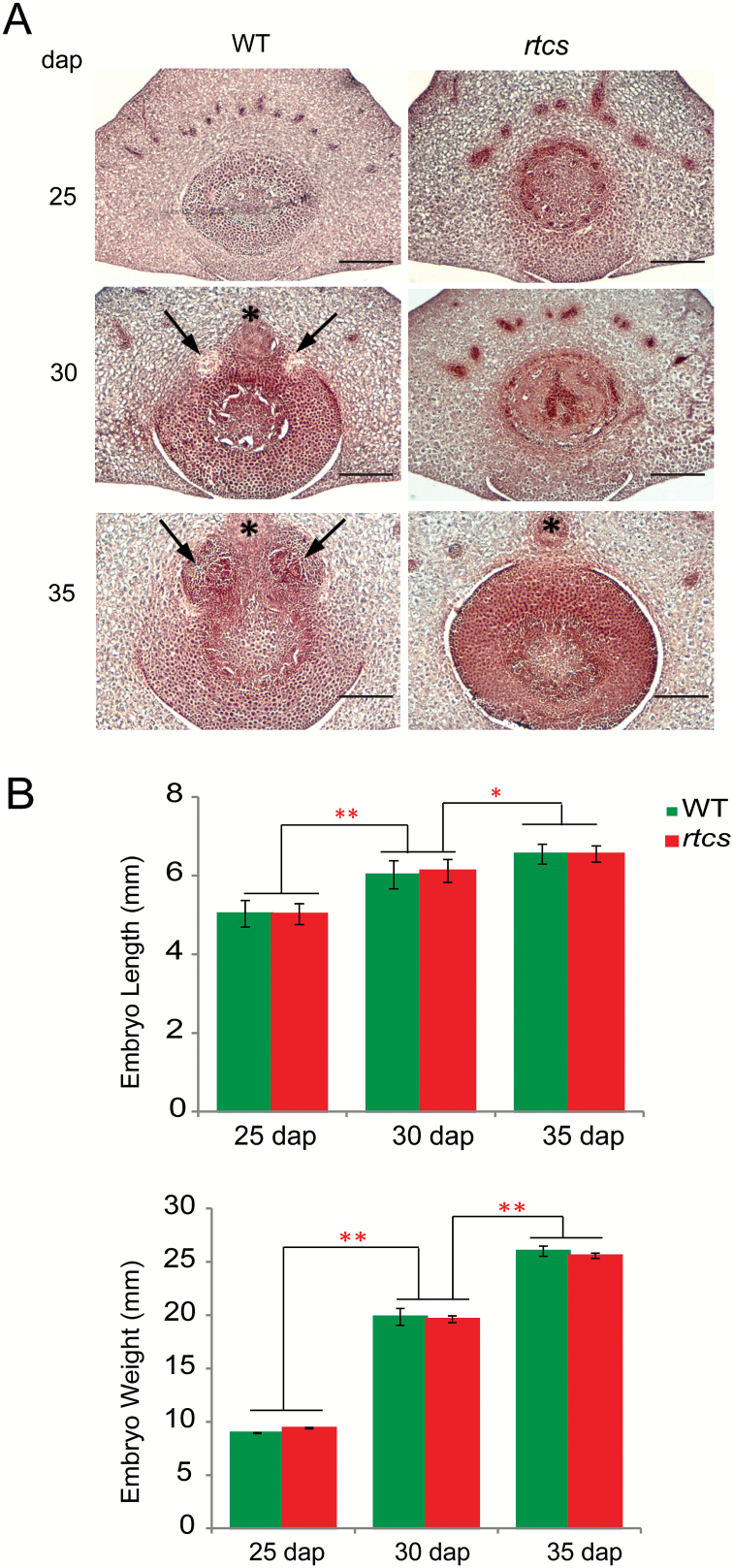
Histological and phenotypic analyses of wild-type (WT) and *rtcs* embryos during formation of seminal root primordia at three developmental stages. (A) Transverse sections of Safranin-/Fast Green-stained WT and *rtcs* embryos 25, 30, and 35 d after pollination (dap). Seminal root primordia are indicated by black arrows. The main strand of the scutellar bundle is indicated by black asterisks. Scale bars are 300 µm. (B) Quantification of embryo length and weight in WT and *rtcs* at 25, 30 and 35 dap. *, *P*<0.01; **, *P*<0.001; *n*=10; error bars indicate ±SD.

To study the process of formation of seminal root primordia, the anatomical structure of wild-type embryos in the region of the scutellar node was examined in transverse sections 25, 30, and 35 d after pollination (dap) and compared with *rtcs* cross-sections at the same developmental stages. At 25 dap, seminal root primordia were neither detectable in cross-sections of wild-type nor in *rtcs* embryos (Fig. 1A). At 30 dap, primordia were initiated in wild-type but not in *rtcs* embryos (Fig. 1A). Finally, at 35 dap wild-type embryos displayed fully developed seminal root primordia while these structures were entirely absent in *rtcs* embryos (Fig. 1A). As expected, embryo length and weight significantly increased during development (Fig. 1B). Embryos of both genotypes that displayed a similar length and weight at each stage (Fig. 1B) were selected for transcriptomic analyses.

### RNA-Seq and transcriptome relationships of wild-type and *rtcs* embryos

Global gene expression profiles of wild-type and *rtcs* embryos at 25, 30, and 35 dap were analyzed using an Illumina HiSeq 4000 platform in four biological replicates per genotype/stage combination. On average, ~32 million 100-bp paired-end reads were obtained per sample (see Supplementary Table S1 at *JXB* online). The sequencing reads were quality checked and ~28 million (88%) of the remaining high-quality reads were mapped to unique positions of the maize reference genome (ZmB73 FefGen_v2; Supplementary Table S1). Finally, after removal of stacked reads that share the same start and end coordinates, ~67% of the remaining reads were mapped uniquely to the 39656 maize high-confidence gene models of the filtered gene set (FGSv2, release 5b.60; Supplementary Table S1).

**Fig. 2.**
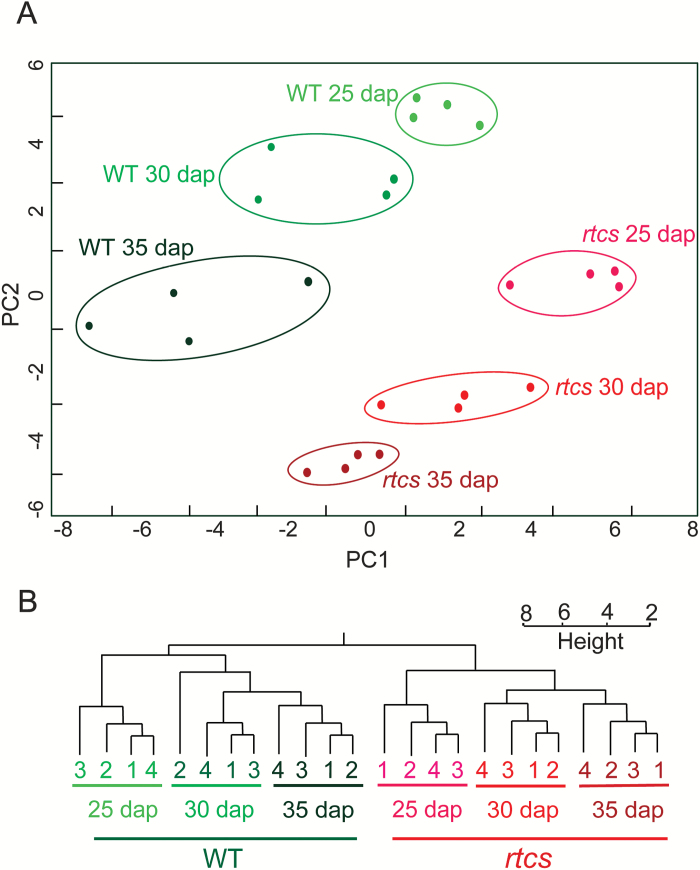
Transcriptome relationships of wild-type and *rtcs* embryos. (A) Principal component analysis (PCA) of wild-type and *rtcs* embryo RNA-Seq samples during development. (B) Hierarchical clustering of the RNA-Seq samples based on Pearson correlation. Height indicates the degree of variance of the *y*-axis.

The relationship of transcriptome samples was assessed by a principal component analysis (PCA) (Fig. 2A) and by hierarchical clustering (Fig. 2B). Both analyses revealed a close relationship of the four biological replicates of each genotype/stage combination. Moreover, the hierarchical clustering analysis revealed that transcriptomes representing the three developmental stages of each of the two genotypes were more closely related to each other than samples of the same developmental stage of different genotypes (Fig. 2B).

### Transcriptome-wide gene activity status in wild-type and *rtcs* embryos

**Fig. 3.**
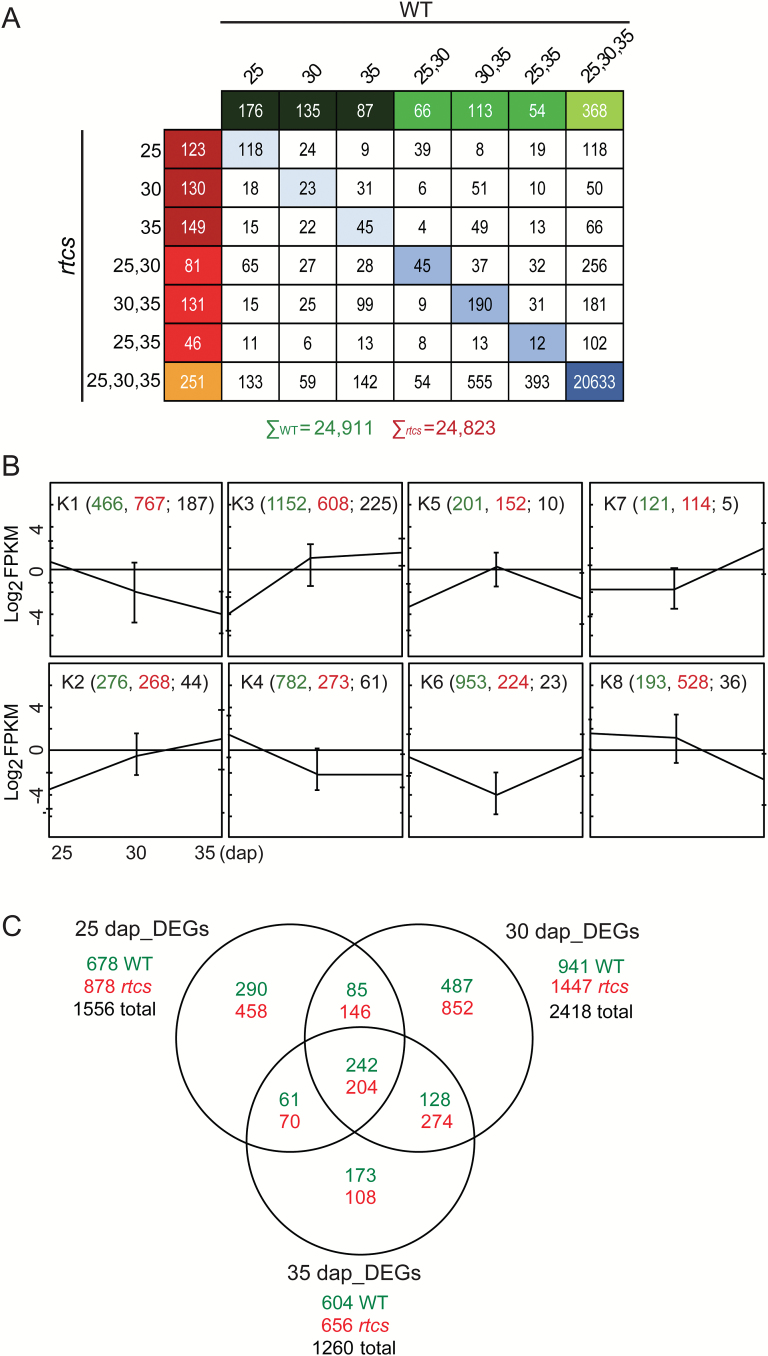
Differential gene expression during embryo development and between wild-type (WT) and *rtcs* embryos. (A) Stage and genotype specificity of gene activity in wild-type and *rtcs* embryos. Cells in green indicate genes exclusively active in wild-type and cells in red indicate genes only active in *rtcs* embryos. Cells in blue indicate genes active in both genotypes at the same stage or stages. (B) K-means clustering showing the pattern changes of gene expression across the three stages in wild-type and *rtcs* embryos. Error bars indicate SD of gene expression in wild-type embryos. Numbers in green and red indicate the genes following the respective pattern in wild-type and *rtcs* embryos, respectively. Numbers in black indicate genes displaying identical patterns in both *rtcs* and wild-type embryos. (C) Venn diagram displaying the number of genes differentially expressed between wild-type and *rtcs* and their overlap during development. For each developmental stage, the total number of differentially expressed genes (black), and the numbers of genes preferential expressed in wild-type (green) and *rtcs* (red) are indicated. DEGs, differentially expressed genes; dap, days after pollination.

A generalized linear mixed model was applied to determine the activity status (active/inactive) of all genes at each of the six genotype/stage combinations (see Methods). In total, 25822 genes (65% of the FGSv2; Fig. 3A; see Supplementary Table S2) were declared active in at least one genotype/stage combination. Wild-type embryos expressed 24911 genes and *rtcs* embryos 24823. A substantial subset of these genes (20633; 80%) was constitutively active in all six genotype/stage combinations, whereas among the genes that were not constitutively active, only between 12 and 190 genes were active at the same developmental stages in both genotypes (blue boxes in Fig. 3A). Notably, hundreds of genes were only active either in embryos of wild-type (Fig. 3A green boxes: 999 genes) or in *rtcs* (Fig. 3A red boxes: 911 genes), suggesting genotype-specific functions of these genes during development. Genes active in only one genotype were functionally annotated based on a set of 3599 classical maize genes with manually curated functions (Schnable *et al*., 2011). This analysis revealed that 92 classical maize genes were exclusively active in wild-type embryos (e.g. the highly abundant ABA signaling gene *ABI3* and the auxin signaling gene *Aux/IAA33*), while 85 classical maize genes were specifically active in *rtcs* embryos. Many of these classical genotype-specific genes are suggested to encode transcription factors (Supplementary Table S3).

### Expression dynamics of wild-type and *rtcs* embryos during development

To explore the dynamics of gene expression during different stages of formation of seminal root primordia in maize embryos, eight patterns of gene expression along the three developmental stages were identified in wild-type and *rtcs* by K-means clustering (Fig. 3B; see Supplementary Table S4). In total, 4144 genes were assigned to the eight clusters (K1 to K8) in wild-type embryos (green numbers in Fig. 3B), while 2934 genes were assigned to *rtcs* embryos (red numbers). This implies less dynamic gene expression patterns in *rtcs* embryos along the three developmental stages than in wild-type embryos. We further compared overlapping gene expression patterns between wild-type and *rtcs* for each of the eight clusters. In total, 591 genes exhibited the same expression pattern in both genotypes (Fig. 3B, black numbers). This suggests that during the formation of seminal root primordia in maize embryos, expression patterns of a large number of genes change in *rtcs* versus wild-type embryos.

Pairwise contrasts were further determined to identify genes differentially expressed between wild-type and *rtcs* embryos (Fig. 3C). Overall, 3526 genes (FDR<5%, |log_2_Fc|≥1) exhibited differential expression at least at one developmental stage in *rtcs* versus wild-type embryos, including 1556 at 25 dap, 2418 at 30 dap, and 1260 at 35 dap (see Supplementary Table S5). In total, 13% (446/3526) of differentially expressed genes were significantly different between wild-type and *rtcs* embryos at all three developmental stages, whereas 67% (2368/3526) genes were differentially expressed at only one developmental stage. Among the differentially expressed genes, 131 genes encoding transcription factors were identified via searches in the maize TF database PlantTFDB v3.0 (http://planttfdb.cbi.pku.edu.cn/; Jin *et al.*, 2013; Supplementary Table S5).

Subsequently, genes with dynamic (Fig. 3B) and differential expression (Fig. 3C) patterns were assigned to MapMan functional categories (Thimm *et al.*, 2004) to identify enriched molecular processes. Enriched functional categories (*P*<0.01) were only found among differentially expressed genes (see Supplementary Table S6). Genes differentially expressed at 25 dap were enriched for the categories ‘hormone’, ‘minor CHO metabolism’, ‘secondary metabolism’, ‘stress’, and ‘redox regulation’. The categories ‘minor CHO metabolism’ and ‘stress’ were enriched among genes differentially expressed at 30 dap. Finally, genes exhibiting differential expression at 35 dap were enriched for ‘minor CHO metabolism’, ‘stress’, and ‘cell wall’.

### Differential expression of genes involved in cell proliferation

**Fig. 4.**
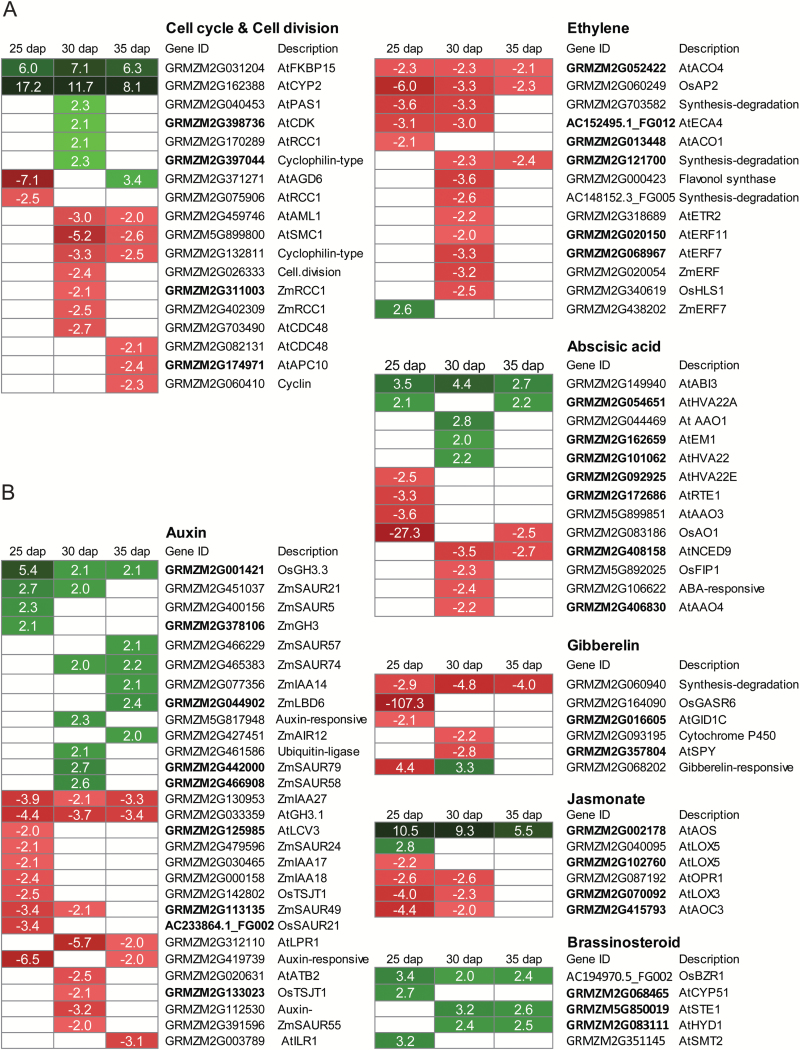
Differentially expressed genes between wild-type and *rtcs* embryos at 25, 30, and 35 d after pollination (dap) involved in cell proliferation (A) and plant hormone metabolism and signaling (B). Values are fold-changes between wild-type and *rtcs*. Green boxes indicate preferential expression in wild-type and red boxes indicate preferential expression in *rtcs*. Blank boxes indicate genes that did not meet the FDR<5% and |log_2_Fc|≥1 criteria. Gene IDs in bold indicate that these genes contain at least one LDB motif within 1 kb upstream of the ATG start codon.

Cell proliferation and differentiation are processes regulating seminal root primordia formation. Accordingly, 18 genes related to cell cycle and cell division exhibited differential expression in at least one developmental stage (Fig. 4A; see Supplementary Table S7). Most genes (13) were differentially expressed at 30 dap, while only four genes were differentially expressed 25 dap, implying active cell division progression from 30 dap onwards. The expression levels of six cell cycle-related genes were up to 17-fold higher in wild-type embryos, including two genes with homology to *AtFKBP15* and *AtCYP2* that were consistently up-regulated >6-fold at all three stages. Decreased expression of cell cycle genes in wild-type embryos, as for instance illustrated by two regulators of cell division cycle protein (CDC48), was also observed.

### Differential expression of genes involved in hormone metabolism and hormone signaling

Phytohormones are essential for the regulation of plant development. Therefore, the functional category ‘hormone metabolism’ enriched among differentially expressed genes at 25 and 30 dap was investigated in more detail. In total, 73 genes differentially expressed between *rtcs* and wild-type embryos are involved in hormone metabolism, hormone signaling, or hormone response (Fig. 4B; see Supplementary Table S7). Among these, increased or decreased expression of 29 genes related to auxin was observed in *rtcs* embryos. All but one of these genes were related to auxin signaling (5) or response (23). For instance, nine genes belonged to the early auxin-responsive *SMALL AUXIN UP RNA* (*SAUR*) gene family. Interestingly, 13 of 14 genes involved in ethylene biosynthesis (6) and signaling (8) were expressed at 2–6-fold higher levels in *rtcs* embryos. Furthermore, expression of five brassinosteroid (BR) -related genes were elevated in the wild-type embryos, including a BR-activated transcription factor gene (similar to *OsBZR1*) and four BR biosynthesis genes. Moreover, 13 genes related to abscisic acid (ABA) biosynthesis (6), signaling (1), and response (6) were differentially expressed. Finally, differential expression of six genes involved in the biosynthesis or signaling of gibberelin (GA) and six genes related to jasmonate (JA) was observed.

### Identification of putative RTCS target genes

**Fig. 5.**
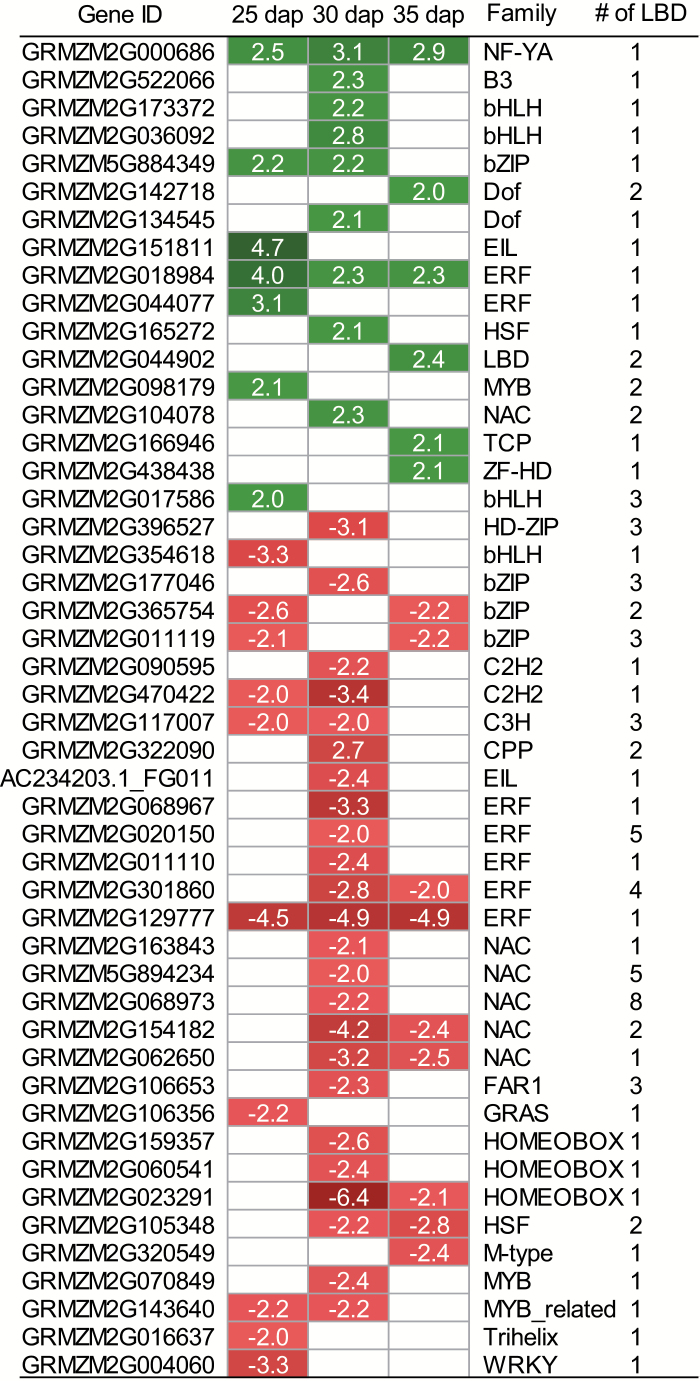
Differentially expressed genes encoding transcription factors between wild-type and *rtcs* embryos at 25, 30, and 35 d after pollination (dap) containing at least one LBD motif. Values in boxes are fold-changes between wild-type and *rtcs* embryos. Green and red boxes indicate preferential expression of genes in wild-type and *rtcs* embryos, respectively. Blank boxes indicate genes that did not meet the FDR<5% and |log_2_Fc|≥1 criteria.

LBD transcription factors such as *rtcs* can bind to LBD promoter motifs of target genes. We therefore specifically examined the abundance of LBD motifs 1 kb upstream of the ATG start codon among 131 differentially expressed genes encoding for transcription factors. This analysis revealed that 48 of 131 (37%) genes contained at least one LBD motif (Fig. 5). Among those, 17 were expressed at higher levels in wild-type embryos while 31 were preferentially expressed in *rtcs*. These putative RTCS target genes mainly belonged to the ERF, NAC, bHLH, bZIP, and HOMEOBOX families of transcription factors. Moreover, the abundance of LBD motifs was examined among differentially expressed genes involved in hormone function. In total, 31 of 73 (43%) genes contained at least one LBD motif (Fig. 4B). Among those, 12 were preferentially expressed in wild-type and 19 in *rtcs* embryos. Finally, at least one LBD motif was found in four of 18 genes associated with cell cycle and division, including preferential expression of two in wild-type embryos and two in *rtcs* embryos (Fig. 4A).

### Evolutionary origin of differentially expressed genes

**Fig. 6.**
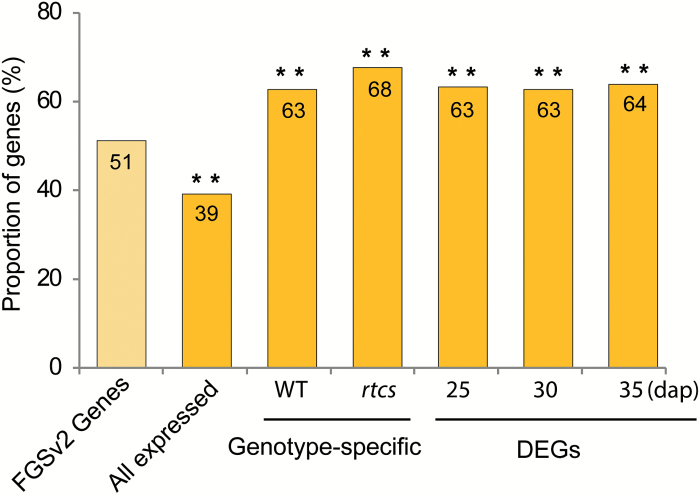
Proportion of syntenic and non-syntenic genes among differential and genotype-specific genes. Significant differences to the proportion of non-syntenic genes in FGSv2 are indicated by red asterisks (*χ* ^2^ test with Yates’ correction). **, *P*<0.001; FGSv2, Maize B73 filtered Gene Set Version 5b.60; DEGs, differentially expressed genes.

Non-syntenic genes emerged after the last whole-genome duplication of maize by individual duplications of syntenic genes. To survey their evolutionary origin, differentially expressed genes were classified into syntenic and non-syntenic genes. The maize filtered gene set (FGSv2) consists of 51% (20291/39656) non-syntenic genes, and 49% (19356/39656) syntenic genes (Schnable *et al.*, 2011). Among all expressed genes in this study, 39% (10117/25822) were of non-syntenic origin (Fig. 6; see Supplementary Table S8). Hence, non-syntenic genes were significantly under-represented among all expressed genes relative to all genes of the maize genome. This suggests that the evolutionary older syntenic genes are expressed more frequently in maize embryos. By contrast, among genes active in only one genotype (see Fig. 3C), 63% (627/999) of wild-type-specific and 68% (616/911) of *rtcs*-specific genes were non-syntenic (Fig. 6; Supplementary Table S8). Similarly, non-snyntenic genes were significantly over-represented among genes differentially expressed between wild-type and *rtcs* embryos (see Fig. 3B) at 25 dap (63%; 984/1556), 30 dap (64%; 1516/2418), and 35 dap (64%; 803/1260) (Fig. 6; Supplementary Table S8). Thus, significant over-representation of the evolutionary younger non-syntenic genes among genes displaying genotype-specific and differential expression reflects a putative involvement of these genes in the RTCS-dependent molecular networks related to seminal root formation.

## Discussion

### RTCS substantially regulates the transcriptomic landscape of maize embryos during seminal root development

Seminal root primordia in maize are formed during embryogenesis in a multi-step process during which cells inside the scutellar node divide (Hochholdinger, 2009). The timing of seminal root initiation depends on the genotype and the growth conditions, and can be observed between 22 and 40 d after pollination (dap) (Sass, 1977; Erdelska and Vidovencova, 1993). In the present study, formation of seminal root primordia was studied in developing homozygous wild-type and *rtcs* embryos using microscopic sections. As described in the Methods section, wild-type and *rtcs* embryos were genetically closely related and highly isogenic. Histological analyses revealed that at 25 dap no seminal root primordia were initiated in wild-type embryos, while early primordia were visible at 30 dap and fully developed primordia were formed at 35 dap under our growth conditions (Fig. 1A). The gene *rtcs* is a major regulator of seminal root initiation as illustrated by the *rtcs* mutant, which does not form any seminal root primordia during embryogenesis (Hetz *et al.*, 1996). Consistently, seminal root primordia were not detected in *rtcs* mutant embryos at any stage between 25 and 35 dap (Fig. 1A). Otherwise, mutant *rtcs* embryos were histologically indistinguishable from their wild-type siblings (Fig. 1B).

These three developmental stages were subsequently selected for transcriptome profiling of *rtcs* versus wild-type embryos by RNA-Seq. Hierarchical clustering revealed that the transcriptomes of wild-type and *rtcs* embryos were more distinct at all three developmental stages than the transcriptomes representing different stages of either genotype (Fig. 2B), suggesting a substantial RTCS-dependent transcriptomic regulation during the formation of seminal root primordia. Similarly, RTCS-dependent transcriptome regulation has been observed during formation of crown root primordia in coleoptilar nodes of maize (Muthreich *et al.*, 2013).

### RTCS-dependent control of cell cycle regulators during formation of seminal root primordia

Formation of lateral organ primordia is driven by the coordinated division of cells and the specification of boundaries of different tissues (Aida and Tasaka, 2006). Hence, formation of lateral root primordia is controlled by the subtle regulation of cell cycle genes in maize pericycle cells (Yu *et al.*, 2015). In this study, functional annotation of differentially expressed genes revealed that RTCS regulates the expression of genes involved in cell cycle control at different stages of formation of seminal root primordia (Fig. 4A). Most of these genes were differentially expressed at 30 dap, suggesting active cell division at this stage. Consistently, seminal root primordia were observed in wild-type embryos from 30 dap onwards (Fig. 1A). Differential regulation of these cell cycle regulators in wild-type versus *rtcs* embryos suggests a role of these genes in the coordination of cell cycle progression in the formation of seminal root primordia. Among these, two genes (*GRMZM2G031204* homologous to *AtFKBP15* and *GRMZM2G162388* homologous to *AtCYP2*) displaying consistently higher expression levels in wild-type embryos are members of the peptidyl-prolyl isomerase (PPIase) gene family essential for regulation of mitosis and cell growth (Lu *et al.*, 2002). Moreover, the regulator of chromosome condensation 1 (RCC1) protein is a critical regulator of the cell cycle and is required for chromatin-induced formation of the mitotic spindle (Carazo-Salas *et al.*, 1999). Accordingly, several genes encoding the RCC1 protein family were differentially expressed.

### RTCS orchestrates phytohormone-dependent transcriptional networks during embryogenesis

The *rtcs* gene encodes an LBD transcription factor that is a central regulator of auxin signaling (Taramino *et al.*, 2007; Majer *et al*., 2012). LBD proteins are essential in lateral organ initiation and patterning, and several members have been demonstrated to be involved in controlling different aspects of root development. For instance, LBD16, LBD18, LBD29, and LBD33 cooperatively regulate lateral root initiation and emergence in Arabidopsis (Okushima *et al.*, 2007; Lee *et al.*, 2009; Feng *et al.*, 2012), RL1 is instrumental in lateral and shoot-borne root formation in rice (Inukai *et al.*, 2005), and RTCS and its paralog RTCL regulate shoot-borne root initiation and elongation in maize (Xu *et al.*, 2015). Differentially expressed genes associated with hormone functions between *rtcs* and wild-type embryos provide an opportunity to study RTCS-dependent regulation of auxin signaling and its interplay with other phytohormones during embryogenesis. In the present study, 29 auxin-related genes (mainly associated with auxin signaling) were differentially expressed (Fig. 4B). Among those, nine early auxin-responsive *SMALL AUXIN UP RNA* (*SAUR*) genes, which have been suggested to play a role in maize root development (Chen *et al.*, 2014), were identified. Moreover, *ZmIAA27* was expressed consistently higher in *rtcs* embryos than in wild-type embryos at all three developmental stages (Fig. 4B). ZmIAA27 is a key component of auxin signaling modules regulating primordia formation of maize tassels and ears (Galli *et al.*, 2015).

Crosstalk between ethylene and auxin has been demonstrated for lateral root initiation in Arabidopsis (Ivanchenko *et al.*, 2008). Moreover, ethylene synthesis involved in stress responses inhibits organ growth (Achard *et al.*, 2006). Elevated expression of a number of ethylene biosynthesis genes was observed in *rtcs* embryos in the present study, including two genes encoding ACC oxidases (GRMZM2G013448: AtACO1, and GRMZM2G052422: AtACO4; Fig. 4B). Ethylene interacts with abscisic acid (ABA) signaling during seed development (Finkelstein *et al.*, 2008). In the present study, a maize homolog (GRMZM2G149940) of the Arabidopsis ABA signaling gene *ABI3* was expressed at significantly higher levels in wild-type embryos at all three stages (Fig. 4B). In Arabidopsis *ABI3* is involved in lateral root development (Brady *et al.*, 2003). The balance between ABA and gibberelic acid (GA) plays an important role in seed development (Finkelstein *et al.*, 2008). Moreover, GA has been demonstrated to control root meristem size (Ubeda-Tomás *et al.*, 2009). In the present study, a GA biosynthesis gene (GRMZM2G164090) homologous to *OsGASR6* was expressed 100-fold higher in *rtcs* than in wild-type embryos at 25 dap (Fig. 4B). *OsGASR6* is highly expressed in rice embryos and is suggested to be a key regulator of GA biosynthesis during seed development (Xue *et al.*, 2012). Jasmonic acid (JA) promotes formation of lateral root primordia by an auxin-dependent mechanism (Raya-González *et al.*, 2012). Moreover, it has been demonstrated that the LBD protein OsIG1 positively regulates the JA biosynthesis gene *EG1* (Zhang *et al.*, 2015). In this study, a JA biosynthesis gene, similar to Arabidopsis *AtAOS*, was expressed 5–11-fold higher in wild-type embryos (Fig. 4B). Brassinosteroid (BR) homeostasis and signaling are necessary for proper organ boundary formation (Gendron *et al.*, 2012). Expression of five BR-related genes was up-regulated in wild-type embryos (Fig. 4B). Moreover, a feedback regulation between the Arabidopsis LBD gene *LOB* and BR-related genes has been demonstrated to limit growth in organ boundaries (Bell *et al.*, 2012).

Taken together, differential expression of diverse hormone-related genes between *rtcs* and wild-type embryos suggests that the RTCS-dependent transcriptional regulation of genes associated with different phytohormones coordinates the complex interplay of molecular networks involved in the formation of seminal root primordia during embryogenesis.

### Potential RTCS target genes during embryogenesis

To date, little is known about direct RTCS target genes in seminal root initiation. It has been demonstrated that RTCS is able to regulate the expression of target genes by binding to LBD promoter motifs (Majer *et al.*, 2012). An *in silico* promoter analysis of genes differentially expressed between wild-type and *rtcs* during embryogenensis revealed that 37% (48/131) of transcription factor genes (Fig. 5) and 43% (31/73) of hormone-related genes contain at least one LBD motif (Figs 4A and 5). Among all differentially expressed genes, only 28% (970/3526) contained at least one LBD motif. Regulatory genes are typically expressed at low levels and are thus difficult to measure in classical microarray studies. As a consequence, in a comparative microarray study of *rtcs* and wild-type coleoptilar nodes, a significantly lower proportion (12%) of putative RTCS target genes was detected (Muthreich *et al.*, 2013) compared with 28% in the present study. In Arabidopsis, it was demonstrated that the DNA-binding affinity of AtLOB to downstream target genes is regulated by interaction with bHLH048 (Husbands *et al.*, 2007). Consistently, several members of the bHLH family were differentially expressed in this presnt study (Fig. 5), suggesting that proteins encoded by these genes could interact with RTCS and control their regulation of downstream genes.

The present study thus provides initial clues on putative RTCS target genes as a basis for further genetic investigations.

### Evolutionary origin of RTCS-regulated genes during embryogenesis

Embryonic seminal roots are formed in maize, whereas they are missing in its close relative sorghum (*Sorghum bicolor*) (Singh *et al.*, 2010). Modern maize was domesticated ~9000 years ago from its ancestor teosinte in the Mexican highlands, and after initial diversification it spread to the lowlands and thus into a completely different ecosystem (Matsuoka *et al.*, 2002). As a consequence of this change, it can be hypothesized that ancient farmers inadvertently selected for genotypes with root architectures that performed better under lowland conditions where, for instance, phosphorus is less available (Lynch and Brown, 2008). One root trait that is associated with the domestication of teosinte into maize is the number of seminal roots. It has been demonstrated that the majority of teosinte accessions (62%) do not form seminal roots (Burton *et al.*, 2013). While the remaining teosinte accessions form a maximum of three seminal roots, maize landraces form up to 11 (Burton *et al.*, 2013) and modern maize varieties up to 13 seminal roots (reviewed in Hochholdinger, 2009).

Analyses of mutants have demonstrated that activity of the genes *rtcs* (Hetz *et al.*, 1996) and *rum1* (Woll *et al.*, 2005) is necessary, albeit not sufficient, to form seminal roots. This is illustrated by the fact that both *rtcs* and *rum1* (Taramino *et al.*, 2007; von Behrens *et al.*, 2011) are syntenic between maize and sorghum, which does not form seminal roots (Woodhouse *et al.*, 2010; Schnable *et al.*, 2011). In the present study, non-syntenic genes were significantly over-represented among differentially expressed genes (Fig. 6). This suggests that genes acting downstream of *rtcs* have mainly been recruited from non-syntenic genes that emerged by individual gene duplications and therefore have no homologs in sorghum. These genes might not have had crucial functions in maize development before their association with RTCS-dependent functions. This is in line with observations that non-syntenic genes might play an essential role in adaptation to environmental challenges (Schnable, 2015). Moreover, the anatomical organization of seminal roots suggests that they are better equipped for efficient soil resource absorption compared with more anciently formed primary and shoot-borne crown roots (Tai *et al.*, 2016).

## Conclusions

Taken together, this study provides first insights into the molecular framework underlying seminal root initiation in maize. Our data suggest that during seminal root evolution the syntenic gene *rtcs* has recruited evolutionary younger non-syntenic genes particularly associated with auxin signaling, transcription factor action, and cell proliferation by transcriptional regulation. Interaction of these genes with RTCS probably resulted in the specification of seminal root primordia and as a consequence contributed to the agronomic success of modern maize.

## Supplementary data

Supplementary data are available at *JXB* online.

Table S1. RNA-Seq output and mapping results.

Table S2. Stage and genotype-specific gene activity patterns.

Table S3. Transcription factors among genotype-specific genes.

Table S4. Genes assigned to eight K-means clusters in wild-type and *rtcs* embryos.

Table S5. The 3526 genes differentially expressed between wild-type and *rtcs* embryos.

Table S6. Assignment of differentially expressed genes to MapMan categories.

Table S7. Genes related to hormone metabolism and cell proliferation differentially expressed between wild-type and *rtcs* embryos.

Table S8. Non-syntenic genotype-specific and differentially expressed genes.

## Supplementary Material

supplementary_table_S1

supplementary_table_S2

supplementary_table_S3

supplementary_table_S4

supplementary_table_S5

supplementary_table_S6

supplementary_table_S7

supplementary_table_S8
